# The US Department of Justice stumbles on visual perception

**DOI:** 10.1073/pnas.2102702118

**Published:** 2021-05-24

**Authors:** Thomas D. Albright

**Affiliations:** ^a^The Salk Institute for Biological Studies, La Jolla, CA 92037

**Keywords:** forensic science, sensory measurement, feature comparison

## Abstract

A large and highly valuable category of forensic evidence consists of patterned impressions created during the perpetration of a crime. These crime scene artifacts, such as fingerprints or tire tracks, offer visual sensory information that is assessed by trained human observers and compared to sensory experiences elicited by model patterns that would have been produced under a hypothesized set of conditions. By means of this “forensic feature comparison,” the observer makes a judgment about whether the evidence and the model are sufficiently similar to support common origin. In light of documented failures of this approach, significant concerns have been raised about its scientific validity. In response to these concerns, the US Department of Justice has made assertions about how forensic examiners perform feature comparison tasks that are not consistent with modern scientific understanding of the processes of sensation and perception. Clarification of these processes highlights new ways of thinking about and improving the accuracy of forensic feature comparison and underscores the vital role of science in achieving justice.

Forensic science is the bread and butter of criminal investigation and prosecution. On the surface of things, it is an incredibly compelling discipline. Artifacts of human activity left without intent or awareness suggest specific action scenarios, implicate specific actors, and sometimes support inferences regarding an actor’s motivation or intent. Indeed, much of the genuine public fascination with—and trust in—forensic science stems from the sense of eavesdropping, from the feeling that we might learn some raw truth that is infinitely more candid simply because the actor was unaware of being watched. All of these fuel righteous indignation against those who would cause criminal offense and gives us the satisfying impression that we have a leg up on the bad guys.

Despite this fanciful optimism and longstanding public support, it has become increasingly clear that forensic practices that rely on human judgment often implicate the wrong people. This form of error frequently has tragic personal and societal consequences, including wrongful conviction and imprisonment. Indeed, thousands of innocent person-years have been spent behind bars for this reason, the majority of these quashed lives being men of color (1).

Many of the problems with this discipline were considered in a landmark 2009 report from the National Academy of Sciences (NAS) (2). This congressionally mandated study identified numerous weaknesses associated with validation, training, and reporting procedures in forensic practice and included detailed recommendations for science-based reform. These recommendations led, most notably, to creation of the short-lived National Commission on Forensic Science, and the National Institute of Standards and Technologies operation known as the Organization of Scientific Area Committees for Forensic Science, and to a variety of grass-roots efforts to improve and standardize forensic practice. In 2015, President Obama asked the President’s Council of Advisors on Science and Technology (PCAST) to further evaluate needs within the forensic science community, the product of which was a 2016 report focusing on a specific subset of forensic practices known as “feature comparison” methods (3).

## Forensic Feature Comparison

Feature comparisons are among the oldest and most commonly employed of forensic methods and are familiar to most by their use for evaluation of visually patterned evidence, such as fingerprints, tool marks, and tire tracks.* Testimony consists of human decisions that are informed by sensory information. The causal origins of latent fingerprints and tool marks, for example, are determined by trained observers who visually compare these crime scene artifacts (the evidence) to patterns that would have been produced under a hypothesized set of conditions (the model, or “exemplar”). The observer makes a perceptual judgment about whether the evidence and the model are sufficiently similar to support common origin.

PCAST observed, as did the NAS before it, that feature comparison methods were problematic, in that “many relied in part on faulty expert testimony from forensic scientists who had told juries incorrectly that similar features in a pair of samples taken from a suspect and from a crime scene (hair, bullets, bitemarks, tire or shoe treads, or other items) implicated defendants in a crime with a high degree of certainty” (3). In response to this harrowing observation, PCAST made a simple point that has long been a foundation of the scientific method and widely acknowledged in other applied sciences, such as medicine and engineering: The legitimacy of any instrument of measurement (and thus its presumed admissibility as a source of evidence in court) depends upon empirical demonstration that the instrument yields an outcome that is correct—that it is valid.

The PCAST report elicited immediate, vociferous, and sustained objections from communities of forensics practitioners and criminal prosecutors (4). Much of the substance of these objections focused on PCAST’s recommendations for broader and more refined testing of the validity of feature comparison techniques. In short, it has been argued that the rigorous validation criteria put forth by PCAST are not sufficiently flexible to deal with the real-world messiness of solving crimes, and that the PCAST members who stand in judgment of the field are not themselves well versed in forensic methods and their application.

Not to be outdone, and in the waning days of a “law and order” administration, the US Department of Justice (DOJ) posted, on January 13, 2021, a detailed critical statement in response to the PCAST report (5). This statement addresses three perceived problems with the report: 1) questionable designation of forensic science within the scientific field of metrology, 2) inflexible criteria for assessing the validity of forensic procedures, and 3) excessive emphasis on black box studies for determining forensic error rates. The second and third of these perceived problems have been reported previously (6) and have been forcefully rebutted by the PCAST Forensic Working Group Chair, Eric Lander (7). I will not dwell on them here. What I will do in the remainder of this perspective is focus on the DOJ’s claim that forensic science does not fall within the scientific discipline of metrology. This may seem like a semantic argument of little consequence, but I maintain that it reflects a longstanding and deep-seated misunderstanding within the forensic science community about how people make decisions. Clarification of the relationship between forensic feature comparison and metrology highlights a biological information-processing approach to forensic practice, which holds much promise for mitigating the tragedy of wrongful conviction.

## Is Forensic Science Metrology?

Metrology is broadly defined as “the science of measurement, embracing both experimental and theoretical determinations at any level of uncertainty in any field of science and technology” (8). It centers on a centuries-old system of definitions and conventions for the standardization of instruments and units of measurement, and has wide applicability in both pure and applied sciences such as engineering, medicine and economics.

Many forensic practices rely upon physical or chemical evaluation of crime scene evidence, which is made possible using machines and measurement protocols designed for that purpose. Forensic toxicology, for example, relies upon machine-based forms of analytical chemistry, such as chromatography and spectrometry, to detect and measure substances (e.g., drugs, toxins) in bodily fluids. Similarly, forensic genotyping employs state-of-the-art tools from molecular biology to isolate and measure sequences of nucleotides for purposes of human identification. These practices indisputably fall within the science of metrology, as measurement is the key to determinations of identity, cause, and criminal responsibility (9).

In its forensic science report (3), PCAST asserted that “feature-comparison methods belong squarely to the discipline of metrology—the science of measurement and its application.” Moreover, because this is so, “science has clear standards for determining whether such methods are reliable” (3). By contrast, the DOJ now argues that feature comparison methods do not qualify as metrology, and thus “the fundamental premise PCAST used to justify its ‘guidance concerning the scientific standards for [the] scientific validity’ of forensic pattern comparison methods is erroneous” (5). Citing the *International Vocabulary of Metrology *(10), the substance of the DOJ’s argument appears to be that feature comparison methods do not measure anything because they do not yield a “quantity value” (5). In particular, the DOJ claims that “forensic pattern comparison methods compare the features/characteristics and overall patterns of a questioned sample to a known source; *they do not measure them*” (my italics). Instead, “patterns are visually analyzed, compared, and evaluated for correspondence or discordance with a known source” (5).

By this argument, the distinction between forensic methods that have long been accepted as belonging to the scientific discipline of metrology and those that fail to qualify is that the former employ man-made devices for measurement and the latter do not, for the “method of comparison is observational” (5). To be clear, the United States Department of Justice, which is not a scientific organization, has made the surprising scientific assertion that visual patterns are not measured by the human brain; rather, they are “visually analyzed” (5). Motivated by the naivete of this claim, and in light of the DOJ’s structural authority, I briefly review here the established scientific understanding of the processes that underlie sensation and perception and give rise to brain-based measurements of sensory stimuli and judgments about their similarity.

## Quantitative Measurement of Stimuli by Biological Senses

Humans and other animals routinely gather information about their environments through the senses, which are patently biological instruments of measurement, discrimination, and classification. Modern neuroscience has revealed how environmental energy of various types—luminous, chemical, and mechanical—is transduced into neuronal energy by specialized receptor systems (11). These neuronal signals are quantified and integrated, enabling organisms to interpret the behaviors of others, to find their way, and to recognize objects of interest, such as food, reproductive partners, and shelter.

The processes by which the senses quantify the physical properties of environmental stimuli became a subject of serious scientific study in the early part of the 19th century (12). The goal of this research was to identify the rules that relate the physical magnitude of a sensory stimulus, such as the weight of an object or the amplitude of a sound, to the magnitude of the conscious perceptual experience elicited by the stimulus. One fundamental insight gained by probing this “psychophysical” relationship was that perceived magnitude can be quantified by units of equal discriminability. The German physiologist/psychologist Ernst Weber conducted carefully controlled experiments in which he determined the smallest perceptible difference between pairs of sensory stimuli, a quantity termed the “difference threshold” or the “just-noticeable difference.” Weber found that the size of this unit of discriminability was proportional to the magnitude of the things being compared. To be perceived, an incremental difference in luminance, for example, must be proportional to absolute luminance. Weber’s student and colleague, Gustav Fechner, generalized this quantitative relationship in what he called *die Massformel*, the measurement formula, which states that the perceived magnitude of a stimulus is proportional to the logarithm of the physical magnitude (13).

As Fechner further developed an experimental psychology based on this psychophysical relationship, he discovered that perceived differences between stimuli could best be quantified not by asking people to report the absolute magnitude of those differences, but rather by asking them to make relative judgments of magnitude. Fechner found that relative judgments can be obtained experimentally using a technique he devised, known as “two-alternative forced choice” (2AFC), in which people are asked to judge the magnitude of a test stimulus relative to a standard: for example, “which is brighter, light A or light B?”

Because of numerous time-varying noise sources—environmental noise, optical noise, transducer noise, and neuronal noise—that impact vision, perceptual reports about the relative brightness of similar stimuli necessarily vary, even when the physical stimuli do not change. Sometimes light A is deemed brighter than light B, and other times vice versa. This quantifiable variation reflects the uncertainty of measurement associated with perceptual experience.

In the simple cases studied by Weber and Fechner, there are dimensions of energy magnitude, such as mechanical pressure and luminance, that define the physical properties of sensory stimuli and can be related to perceived intensity. Many stimuli differ from one another, however, along dimensions that do not correspond directly to stimulus magnitude, such as the shape or or texture of a pattern. In cases where stimulus differences manifest along complex combinations of such dimensions, where there is no simple scale that captures the physical differences between stimuli, the stimulus properties are termed “nominal.” The DOJ maintains that the nominal properties of forensic patterns cannot be measured as scalar quantities by a human observer: “Measurement, however, does not apply to ‘nominal’ properties—features of a phenomenon, body, or substance that have no magnitude” (5).

This argument is flawed for two reasons. First, nominal properties of sensory stimuli are, by definition, categorical assignments expressed in words, but those words do not correspond to human discriminability. There is, for example, an ∼75-nm range of wavelengths of light that most people would call green, but they can readily discriminate 5-nm differences in wavelength within that range (14). More generally, any two visual patterns of arbitrary dimensional complexity may be best described in words—such as loops, whorls, and arches, or boubas and kikis (15)—but their discriminability can always be quantified along a scalar dimension of perceptual similarity. In forensic feature comparisons, it is the expert’s ability to discriminate between pairs of sensory stimuli that matters, not what the stimuli are called.

Second, the DOJ asserts, again citing the *International Vocabulary of Metrology*, that measurement (and thus metrology) cannot apply to properties of sensory stimuli that have no magnitude. What sensory systems actually measure is information (of which energy magnitude is one type), for it is information that enables us to interact effectively with the world we live in. Contrary to the DOJ’s claim, sensory information varies along any number of scalar dimensions that are readily measured by the human visual system (16).

## Discrimination of Forensic Patterns by Biological Senses

Building on this basic understanding of sensory measurement, we can illustrate the process by which forensic examiners make decisions when confronted with feature comparison tasks. In the simplest case, the sensory stimuli consist of two visual patterns, such as pairs of fingerprints or tire tracks. One pattern (the evidence) is collected from the crime scene and the other (the exemplar) drawn from a database of previously recorded patterns. The forensic instrument (the observer’s brain) performs two critical operations on these patterns. The first operation is sensory measurement. The two visual patterns are measured independently, according to the processes described above, and the results are compared to yield a compound measure of perceptual similarity. That similarity measure ranges continuously from low to high and serves as the “decision variable.” This scalar variable is the input to the second operation, which is evaluation of the similarity measure relative to the instrument’s standard, known as the “decision criterion.” This is a classic signal detection problem in which similarity values that exceed the criterion meet the requirement for a same-source designation; those that do not are dismissed as having originated from different sources.

Although the forensic instruments are materially different—one biological and the other machine—the operations performed by a pattern examiner’s brain and a chromatograph for analytical chemistry are functionally identical. Both of these instruments measure the similarity of the evidence relative to a known-source sample, apply a decision criterion to that similarity measure, and render a categorical decision. The biological instrument differs from the machine, however, in one crucial way: Neither the decision variable nor the criterion is apparent to anyone observing the outcome. There are no LED panel displays or graphical readouts of the underlying “quantity values” (5). This inscrutability is the proximal cause of the DOJ’s errant reasoning. To those uninformed about how sensory systems actually work, the process of feature comparison looks as though nothing has actually been measured and the result is attributed to unaccountable “visual analyses.”

Discrimination of things measured by the senses is, of course, not unique to forensic science. This is what sensory systems do best. Application of the DOJ’s reasoning to other domains of human discriminability lays bare its full absurdity, since it argues that the infinite variety of human sensory decisions—such as wine and food tasting, matching colors of paint, distinguishing the scents of flowers, identifying the provenance of a violin by its timbre, or recognizing the face of a friend in a crowd—indeed, entire disciplines that depend upon human decision-making, such as medical practice, are not based on quantitative measurement. People can, of course, perform all of these tasks well and do so because of the fine measurement abilities of biological senses.

## Estimating the Accuracy of Forensic Decisions

A critical quality of any forensic decision is its accuracy. All else being equal, accuracy is tied to the strength of the decision variable, which for many forensic tasks is a measure of the similarity between evidence and exemplar. In the case of the chromatograph, the strength of similarity is a machine readout that is readily accessible to the recipient of the decision. That strength is the foundation for machine discriminability and thus usefully predictive of decision accuracy.

By contrast, because the decision variable and criterion are covert in forensic feature comparison by human observers, a given categorical decision reveals nothing about the strength of the evidence or the accuracy of the decision. While we might expect that the combination of strong similarity and a stringent criterion is more likely to yield an accurate decision than is weak similarity and a loose criterion, the recipient of the decision is not in a position to know which is true.^†^

Recent work on human decision-making in the context of eyewitness identification has focused on the possibility that confidence judgments can overcome this inverse problem by providing proxy insight into the underlying decision variables and criteria (17–19). Specifically, confidence in a decision is correlated with the decision criterion applied and thus, indirectly, the strength of the decision variable.^‡^ By this relationship, confidence in a forensic decision made by a human observer is an overt scalar measure of the underlying strength of perceptual similarity, which is further evidence that human senses yield quantitative measurements. As for machine-based forensics, this measured quantity is usefully predictive of decision accuracy (19).

Another behavioral tool for estimating strengths of the underlying similarity measures is a variant of Fechner’s 2AFC task, developed in the early 20th century by the American psychologist Louis Thurstone (20). Thurstone’s insight was that relative judgments of sensory stimuli could be obtained by reference to an arbitrary standard. In this case, people are simply asked to report which of two stimuli is more similar to the standard (“Which cheesecake tastes more like your favorite?”; a common type of question in scientific studies of taste preferences) (see refs. 21 and 22). The advantage of this approach is that it does not require that the stimuli themselves be physically quantified, which is difficult, if not impossible, for complex stimuli that vary along multiple sensory dimensions. (There is no simple physical metric that corresponds to better-tasting cheesecake.) Moreover, by this means a large set of stimuli, such as candidate exemplar fingerprints, can be “perceptually scaled” in a form that quantifies their respective perceptual similarities to a latent print from the crime scene. The strengths of these measures convey the probability that one of them is a correct match.

## Toward a Psychophysics of Forensic Feature Comparison

The problem of forensic feature comparison necessarily requires quantitative measurement of the patterns under consideration, followed by measurement of their perceptual similarity and application of a decision criterion for “sufficiently similar.” As reviewed above, the human brain has these capabilities. The finely developed concepts and methods of psychophysics are tailor-made for this application and provide many insights that can further improve forensic practice. Any student of psychophysics will note, for example, that it is impossible to meaningfully interpret the classification decision made by a forensic examiner without knowledge of the uncertainty associated with measurement of the sensory patterns. Psychophysics offers an established empirical approach to this problem: Uncertainty can be assessed from repeated psychophysical measures, which combined with signal detection analysis yields a bias-free index of pattern discriminability in the face of measurement noise (23).

A recent study of forensic fingerprint examination demonstrates the utility of this approach (24). Using the psychophysical methods reviewed above, this study evaluated operating characteristics of examiners tasked with comparing many pairs of fingerprints. In this framework, forensic examiners explicitly serve as human measurement devices, and their perceptual reports reflect brain-based discriminations of pattern similarity. Results revealed that “fingerprint experts possess impressive pattern matching abilities that may rival those of medical diagnosticians” and compare favorably to machine-based metrics analyzed using a similar signal detection approach (25).

My laboratory applied a similar psychophysical approach to the forensic problem of eyewitness identification in police lineups (26). The result was a scaling of the perceptual similarity of all lineup faces relative to the witness’ memory of the culprit, which serve as estimates of the underlying strengths of recognition memory for each face. We modeled the probability distributions of these estimates and used a signal detection procedure to calculate in precise quantitative terms each witness’ ability to discriminate the culprit from an innocent suspect.

## Summary and Conclusions

Alarmed by the DOJ’s curious assertion that forensic visual patterns are not measured by the human brain, rather they are “visually analyzed” and the “method of comparison is observational,” I’ve briefly reviewed scientific understanding of the processes that underlie sensation and perception. To wit, biological senses employed by human observers measure and discriminate the physical properties of sensory stimuli by simple and well-established rules. This understanding encourages new ways of thinking about and improving the accuracy of forensic feature comparison and thereby limiting the scourge of wrongful conviction.

Yes, of course this is true: Many extraordinary and everyday feats of human activity—playing a fretless stringed instrument, hitting a baseball, threading a needle, shooting pool and, indeed, assessing the similarity of forensic patterns—reflect the same processes of sensory measurement and comparative judgment, all of which affirms human observers as instruments of measurement. Forensic science thus surely qualifies as metrology.

But that isn’t really the point. The future of forensic science requires that those who wield power talk sensibly about how people make decisions, that the legal apparatus of our society acknowledge and embrace relevant scientific truths and methods. Only by these rational means can we achieve justice for all.

## Data Availability

There are no data underlying this work.

## Footnotes

*Most forensic pattern evidence comes in a visible form and thus most of the discussion herein focuses on the visual system. There are, nonetheless, forms of pattern evidence that present to other sensory modalities (e.g. voice recognition by the auditory system). The same underlying principles apply.

^†^This is a classic “inverse problem,” in that there is not sufficient information in the product of a process to infer the values of the parameters that gave rise to it.

^‡^This correlation holds under "pristine" conditions, in which the decider has not been exposed to other sources of information.
